# Literature review, report, and analysis of genotype and clinical phenotype of a rare case of ulnar-mammary syndrome

**DOI:** 10.3389/fped.2023.1052931

**Published:** 2023-03-03

**Authors:** Xiwen Zhang, Lifen Chen, Lin Li, Jingjing An, Qinyu He, Xuelei Zhang, Wenli Lu, Yuan Xiao, Zhiya Dong

**Affiliations:** Department of Pediatrics, Ruijin Hospital, School of Medicine, Shanghai Jiao Tong University, Shanghai, China

**Keywords:** ulnar-mammary syndrome, TBX3 gene, genotype, phenotype, inherited metabolic diseases (IMD)

## Abstract

**Objective:**

The clinical characteristics of Ulnar-mammary syndrome (UMS) caused by mutations in *TBX3* (T-Box transcription factor 3) were studied and the correlation between genotype and clinical phenotype were analyzed to improve awareness and early diagnosis of the disease.

**Methods:**

The clinical data of a boy aged 13 years and 5 months with left forearm deformity and growth retardation as the main features were analyzed. Genomic exon detection was performed, and the results were verified by Sanger sequencing. Simultaneously, we performed literature review to analyze the correlation between clinical phenotypes and genotypes.

**Results:**

The clinical manifestations in the child were short stature, ulnar hypoplasia of the forearm, hypohidrosis, retracted nipple, micropenis, and cryptorchidism. Laboratory examination revealed hyperthyroidism, growth hormone deficiency, and hypogonadotropic hypogonadism. Imaging results displayed delayed bone age, small pituitary gland, and persistence of Rathke's cleft cyst. The results of the exome sequencing revealed the deletion of AGA at positions 1121–1,124 of *TBX3*, which resulted in a frameshift mutation (c.1121–1124del AGAG; pGlu374fs). According to the American College of Medical Genetics (ACMG) assessment, the mutation is a pathogenic variant. A definitive diagnosis of UMS was made on the basis of the clinical phenotype of the patient. The Chinese and English literature were reviewed to analyze the correlation between *TBX3* genotype and clinical phenotype.

**Conclusion:**

UMS is a rare hereditary disease caused by mutations in *TBX3*. There is significant clinical heterogeneity associated with the variants of this gene. To our knowledge, this mutation site in *TBX3* has been reported for the first time, thereby expanding the mutation spectrum of this gene.

## Background

Ulnar-mammary syndrome (UMS) (OMIM181450) is an extremely rare autosomal dominant disorder. To date, approximately 128 cases have been reported worldwide, and only 2 cases have been reported in China (including this case). In 1997, Bamshad et al. (1) confirmed that UMS is caused by a mutation in *TBX3*. This gene mainly controls the development of the limbs, mammary glands, apocrine glands, and reproductive organs. Therefore, the symptoms of UMS are highly variable. They manifest as limb development defects, apocrine gland and mammary gland dysplasia, reproductive system dysplasia, short stature, and abnormal pituitary structure. Owing to the rarity of the disease and the heterogeneity of its symptoms, it is easily misdiagnosed. This article reports the clinical data and genetic analysis results of a child diagnosed with UMS at the Pediatrics Department of Ruijin Hospital Affiliated to Shanghai Jiaotong University, China, in order to improve physicians’ knowledge and avoid delayed or missed diagnosis of the disease.

### Case presentation

The patient was boy aged 13 years and 5 months, born with a deformity of the left forearm. After the age of 3, his stature was shorter than that of children of the same age; his penis was always shorter than that of children of the same age and did not develop in the past two years; and he was born with bilateral cryptorchidism and underwent bilateral orchidectomy. The patient had been sweating less since childhood, and his intelligence was normal.

The patient was G1P1, delivered by caesarean section at term, with a birth weight of 3400 g and birth length of 50 cm. He was artificial feeding. His motor development was consistent with that of other children of the same age and sex. The parents are not consanguineous, and the mother was healthy during pregnancy. The father is 170 cm tall and in good health; the mother is 158 cm tall and has deformities in the distal segments of the little fingers of both hands, but with no other diseases.

**Figure 1:**
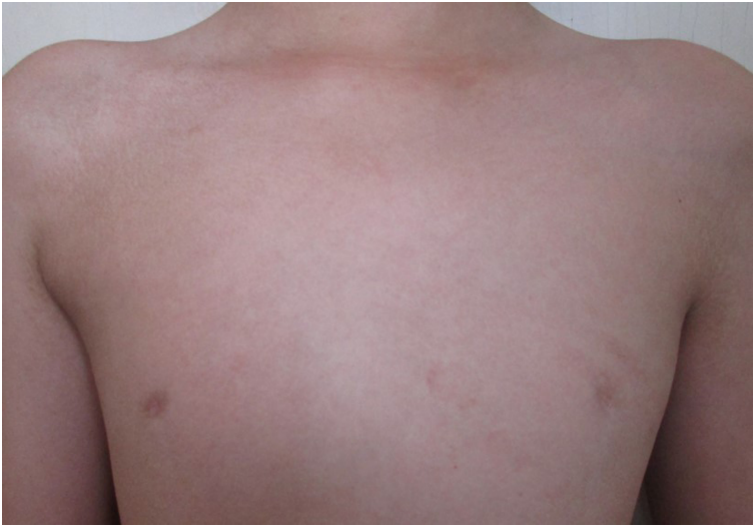
The wide nipple gap and inverted nipples observed in the patient.

**Figure 2:**
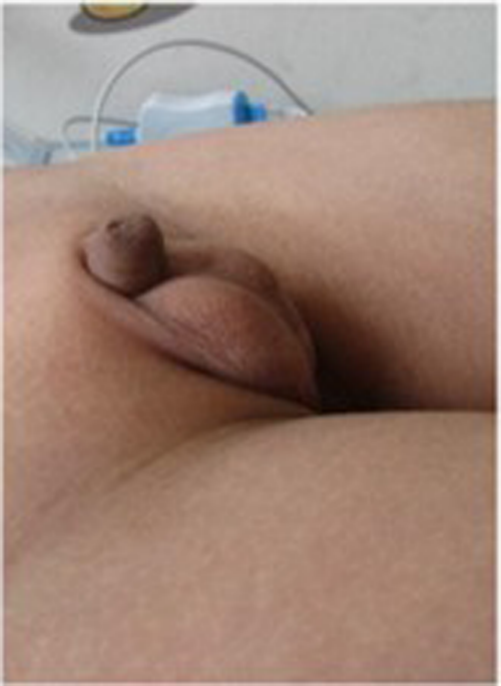
The small penis and testicles observed in the patient.

**Figure 3:**
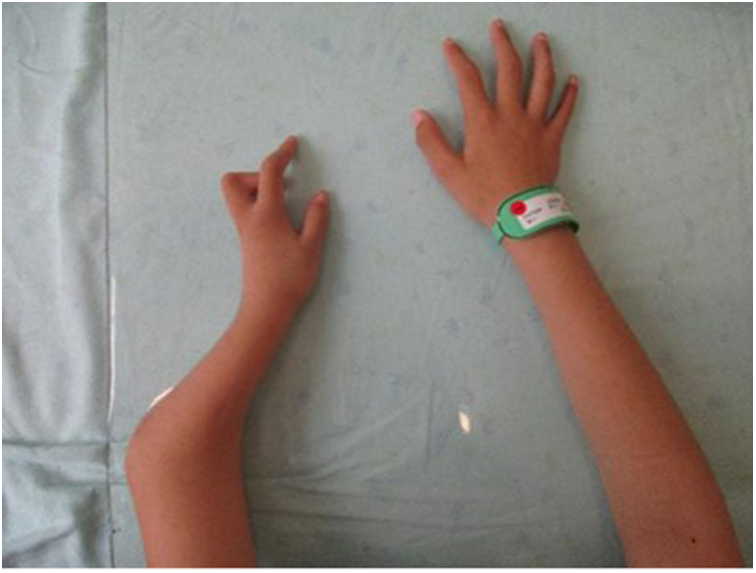
Deformity of the left forearm.

**Figure 4:**
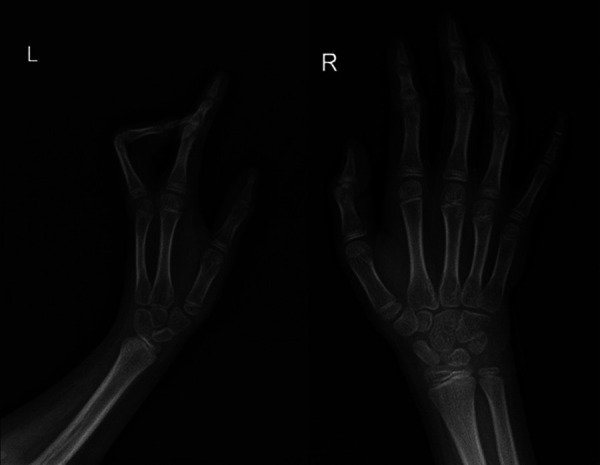
x-ray showing the absence of the left ulna, 4th and 5th metacarpals, and phalanx of the left hand.

The child's height, weight, and body mass index (BMI) were 144 cm (−2.4SD), 33.8 kg, and 16.3 kg/m^2^, respectively. The nipples were wide apart and retracted (Figure 1), penis was 2 cm × 1 cm in size, and testicles were 1 ml in volume (Figure 2). His 4th and 5th metacarpals and phalanges of the left hand were absent, and the left ulna was also absent (Figures 3, 4). The body was well-proportioned and no noticeable abnormalities were observed on the face or detected in the heart, lungs, or abdomen.

Laboratory routine blood, liver function, and renal function tests were normal. The other results are shown in the Table 1 below. All the detection was performed using chemiluminescence.

**Table 1:** Endocrine hormones of the patient.

Tests	Result	Reference range	Measurement
IGF-1	186.0	183–850	ng/ml
IGFBP3	4.2	3.1–9.5	ng/ml
Growth Hormone	1.4	0.1–10	ng/ml
Growth Hormone peak*	6.4	> 10	ng/ml
Luteinizing Hormone	< 0.07	0.3–6	mIU/ml
Luteinizing Hormone peak**	0.8		mIU/ml
Follicle-stimulating Hormone	1.2	0–5	mIU/ml
Follicle-stimulating Hormone peak**	6.4		mIU/ml
Testosterone	< 0.08	3–10	ng/ml
Testosterone peak***	3.7		ng/ml
Dihydrotestosterone	25.0	14–77	pg/ml
Dihydrotestosterone peak***	157.0		pg/ml
Androgens	0.2	0.3–2.6	ng/ml
Androgens peak***	0.4		ng/ml
Anti-mullerian Hormone	> 24.6		ng/ml
Inhibin-B	66.9	56–113	pg/ml
Triiodothyronine	2.7	0.9–2.4	nmol/L
Thyroxine	167.3	62.7–151	nmol/L
Free-triiodothyronine	9.1	2.6–5.7	pmol/L
Free-thyroxine	29.4	9–19	pmol/L
Thyroid Stimulating Hormone	0.0001	0.04–5	uIU/ml
Thyroglobulin Antibodies	80.1	< 4.1	IU/ml
Thyroid Peroxidase Antibody	0.06	< 5.6	IU/ml
Thyrotropin Receptor Antibody	0.3	< 1.75	IU/L

* After clonidine and arginine stimulation test.

** After GnRH stimulation test.

*** After hCG stimulation test.

The electrocardiogram showed sinus tachycardia. The bone age was estimated to be 11 years (actual age was 13 years and 5 months) based on Greulich and Pyle's radiographic atlas.. Furthermore, a pituitary magnetic resonance imaging (MRI) plain scan showed small adenohypophysis and the presence of Rathke's cleft cyst.

### Genome sequencing

Peripheral venous blood was collected from the proband and his parents, Genomic DNA was extracted using the MyGenostics Genomic DNA Purification kit per manufacturer's protocol. After fragmentation and end-repair of genomic DNA from the proband, adapter ligation as well as PCR enrichment were performed following the manufacturer's protocol. Exons and the flanking regions of all known genes were capture. The library was sequenced by an Illumina HiSeq 4,000 sequencer with pair-end 150 mode. Bioinformatics analysis was performed in the framework of bcbio-nextgen. After trimming adapters and low-quality reads, cleaned reads were aligned to the human reference genome (version GRCh37). We filtered out variants with minor allele frequency  > 0.05 and at least 2,000 alleles were observed in any general continental population in gnomAD database. Pathogenicity was predicted using Polyphen2 and MutationTaster.

Sanger sequencing was used to verify the variants in the proband revealed by WES, and to test the co-segregation of variants in the kindred.

**Figure 5:**
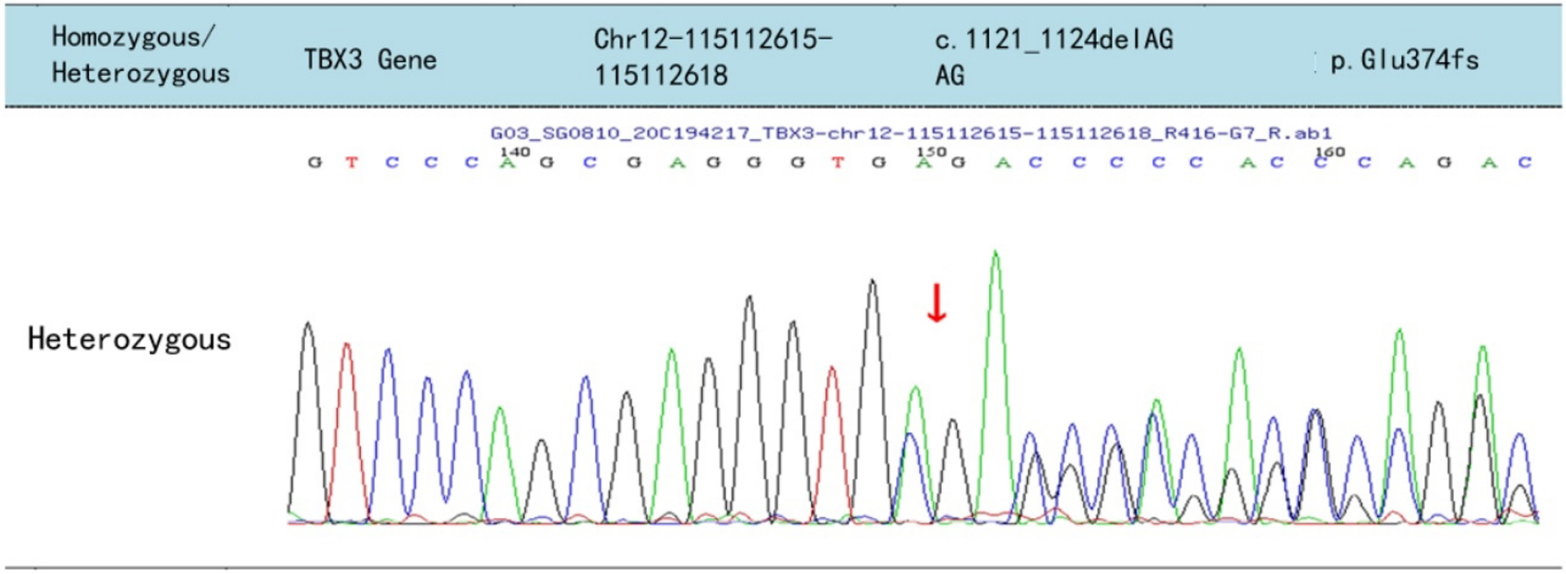
Sequencing results of the *TBX3* of the patient, the arrows indicate the deletion of AGAG from positions 1,121 to 1,124 in the cDNA, resulting in frameshift mutation of the glutamic acid at position 374 of the TBX3 protein.

A heterozygous *TBX3* variant NM_016569.3: c.1121–1124del AGAG (*p*.Glu374fs) was identified (Figure 5). The score was PVS1 + PS4, which is regarded as a pathogenic mutation according to the American College of Medical Genetics guidelines (2). Sanger sequencing confirmed that the variant was of maternal origin.

### Literature search results

Using “(Ulnar-breast syndrome) or (Ulnar-mammary syndrome) or (Schinzel syndrome) or (Ulnar-mammary syndrome of Pallister)” as the search term, we searched Chinese databases such as CNKI, Wanfang, and VIP, and 1 relevant Chinese article was obtained. One case was reported with short stature, deformed face, absent nipple, and polydactyly. We further searched the Pubmed database using the terms “(Ulnar-mammary syndrome) or (UMS) or (Schinzel syndrome) or (Ulnar-mammary syndrome of Pallister) and (TBX3)”, from 1976 to 2022, and 82 relevant articles in English were retrieved. The present study analyzes the genotype and phenotype of patients in combination with the literature to improve the awareness of the disease and the rate of early diagnosis.

## Discussion

UMS is an autosomal dominant genetic disease with an extremely low incidence rate. 128cases of UMS have been reported worldwide, and only two cases, including this case, have been reported in China. UMS is characterized by defects in the limbs, secretory glands, genitalia, growth, and development. The patients have abnormal limb development, such as ulnar hypoplasia or absence, and ulnar finger defects. Other clinical symptoms include apocrine gland and mammary gland abnormalities, including less axillary sweating, mammary gland dysplasia; reproductive system abnormalities, including delayed puberty, micropenis, and cryptorchidism in males, and hymen atresia in females; growth abnormalities, including short stature and growth hormone deficiency; pituitary structural abnormalities, including thin pituitary and pituitary stalk, and hypoplasia of the adenohypophysis; cardiac system abnormalities, including pre-excitation syndrome, arrhythmia, supraventricular tachycardia, and structural deformity of the heart; abnormal eye development; and mental retardation (3). In this case, the patient presented with a syndrome of multisystem involvement, such as absence of the ulna and two fingers on the ulnar side of the left forearm, retracted nipple, micropenis, cryptorchidism, short stature (growth hormone deficiency), underdeveloped adenohypophysis, and Rathke's cleft cyst. The patient's symptoms were consistent with those of UMS.

Owing to the rarity of the disease and the heterogeneity of its symptoms, it is easily misdiagnosed as the diseases with developmental dysplasia of limbs, breasts, etc. For example, Holt-oram Syndrome (HOS), Scalp-ear-nipple syndrome (SENS) and Acro-dermato-ungual-lacrimal-tooth syndrome (ADULT syndrome). Congenital cardiac malformations were more common in patients with HOS than UMS. In addition, HOS doesn’t show symptoms such as sexual developmental delay, apocrine dysplasia and pituitary growth hormone deficiency (4). Although SENS also has symptoms of mammary gland hypoplasia, the main manifestations are congenital scalp hypoplasia and external ear deformity. What's more, there is no sexual development delay and pituitary growth hormone deficiency in SENS (5). ADULT syndrome, like UMS, may present with decreased sweating, acral dysplasia and mammary gland dysplasia. However, acrodysplasia in ADULT syndrome is often manifested as dysplasia of fingers and/or toes instead of radius or ulnar defects. Symptoms such as sexual developmental delay and growth hormone deficiency did not appear in these patients (6).

The pathogenesis of UMS involves TBX3 protein haploinsufficiency caused by mutations in *TBX3*. Studies have shown that the expression product of *TBX3* is a transcription factor that promotes the formation of the embryonic endoderm lineage (7, 8). TBX3 plays a predominant role in self-renewal and multilineage differentiation of embryonic stem cells. This ensures that the differentiation and development of various organs and tissues during the embryonic period can proceed normally by maintaining normal embryonic stem cells (9). When *TBX3* is defective, its binding to DNA is reduced, resulting in loss of function of TBX3 in organ and limb development. As a result, most organs and tissues associated with the endoderm and mesoderm are defective to varying degrees. Mouse studies have shown that homozygous mutations in *TBX3* cause embryonic lethality, whereas heterozygous mutations cause UMS, and the gene mutation has incomplete penetrance (9). Consequently, there is considerable heterogeneity in the clinical presentation of UMS; even among family members with the same genetic mutation, clinical presentation differs.

Abnormal limb development is a major symptom of UMS. Literature shows that UMS has various upper limb deformities, including ulnar hypoplasia, ulnar finger loss, polydactyly, and little finger rigidity (10, 11). In humans, embryonic limb bud growth and formation depend on three key signaling centers; the apical ectoderm ridge, dorsal ectoderm, and polarized active zone. TBX3 is mainly expressed in apical ectodermal ridges, regulates normal limb bud development, and contributes to the determination of limb axial positioning (12). Mouse studies have shown that the extent of limb defects depends on the time of turning off *TBX3* expression during embryonic development, i.e., early-stage cessation leads to initial failure and deformity of the limb, whereas late-stage termination leads to loss of fingers (13). In addition, the condition of patients with UMS is related to the degree of decreased TBX3 expression in the tissues (14). In this case, the patient's left ulna and the 4th and 5th metacarpals and phalanges of the left hand were absent. Therefore, we hypothesize that insufficient levels of TBX3 affect the limbs during middle and late embryonic development. In this patient, even though the proximal limbs developed normally, the patient presents with bone loss in the middle and distal upper limbs.

TBX3 is one of the earliest markers of embryonic mammary epithelial development. Mouse studies have shown that TBX3 induces mammary bud development (12, 15) and maintains growth of mammary epithelia (16). Haploinsufficiency due to heterozygous mutations in *TBX3* results in failure of nipple and mammary ductal tree development (17). In previously reported cases, most UMS patients had various degrees of mammary gland dysplasia, including complete absence of breasts, retraction of nipples, and loss of areola pigmentation (10, 18). In this case, the patient presented with bilateral nipple retraction, and his mother, with the same genetic variant, also displayed bilateral nipple retraction, which is consistent with the clinical manifestations and heredity of UMS. The mild phenotype of breast abnormalities in children may be related to the *TBX3* genotype. It has been suggested that when the T-box domain of the gene is intact, the transcription factor has residual activity and the phenotype is relatively mild (19). In this case, the mutation was located at the 3′ end, downstream of the T-box domain, and close to the C-terminus. The upstream and internal structures of the T-box domain were intact, so the child and mother did not have serious symptoms, such as missing nipples and breasts.

Most children with UMS have hypogonadism and delayed puberty, which may be due to several reasons. First, pluripotent stem cells, induced by TBX3 and other factors promote gonadal development (20). Second, TBX3 is expressed in both the infundibulum and the precursor Rathke's sac of the anterior pituitary (21). It plays a substantial role in the formation of the pituitary infundibulum (22) and may be involved in the development of the hypothalamic-pituitary axis together with other genes (23). Therefore, *TBX3* mutations can cause pituitary gland hypoplasia, resulting in hypogonadotropic hypogonadism (HH). Schinzel et al. (24) (1987) and Galazzi et al. (25) (2018) have reported HH in children with UMS. The patient in this case was 13 years old and still had a small penis, small testis, and low sex hormone levels. The head MRI showed that his pituitary gland was underdeveloped. Therefore, we considered the presence of HH in this patient.

*TBX3* is mainly expressed in the upper extremities, so the bone loss caused by the mutation mainly manifests in the upper limbs, whereas the lower limbs and spine are basically normal (1, 26). Height is largely determined by the development of the spine and the long bones of the lower extremities. Therefore, theoretically, the height of patients with UMS is not directly affected by mutations in this gene. However, short stature has also been reported in previous UMS case reports (approximately 16%) (27). In 2018, Galazzi et al. (25) reported a case of a 15-year-old boy with UMS and short stature. His blood IGF-1 level was low at 182 ng/ml (reference value 152–324 ng/ml) and he did not undergo tests for growth hormone secretion. Brain MRI revealed hypoplasia of the anterior pituitary gland with a normal pituitary stalk. As discussed before, *TBX3* mutations may lead to hypoplasia of the pituitary gland, which as a result secretes insufficient growth hormone, thereby affecting the height of children. The height of the patient in this case was 144 cm (−2.43SD), IGF-1 levels were 186 ng/ml (<−2SD), and the peak GH excitation was 6.39 ng/ml. The patient was diagnosed with GHD, combined with pituitary MRI results, which suggested that short stature may be related to pituitary dysplasia.

During the early stage of cardiac development, TBX3 is expressed in the myocardial atrioventricular canal that separates the ventricular and non-ventricular muscles and is involved in the development of the ventricular conduction system (28). Therefore, TBX3-deficient embryos may develop ventricular septal defects, outflow tract malformations, and arrhythmias (13, 29). However, cardiac abnormalities associated with UMS have rarely been reported. In 2005, VascoMeneghini et al. (19) reported a case of a boy with UMS who had a ventricular septal defect and pulmonary artery stenosis. In 2009, Linden et al (30). reported another case of UMS in a child with a ventricular septal defect and paroxysmal supraventricular tachycardia. In the present case, echocardiography showed no abnormalities, but the electrocardiogram showed sinus tachycardia. However, considering that the child had hyperthyroidism, the test was not specific. Therefore, changes in the heart rate of the child need to be monitored after the thyroid index is normal. We will monitor heart rate changes in children after the thyroid function is normal. Cardiac disease is rarely reported in association with UMS. In addition to the tissue's sensitivity to TBX3 levels, cardiac structure and conduction tissue can be regenerated and reorganized to replace the function of TBX3-deficient cells (31). Therefore, most patients do not have severe heart disease.

Mutations in *TBX3* affect the development of TSH-secreting cells. However, there are other factors that play a critical role in the development of TSH-secreting cell populations (21, 32). Hence, thyroid disorders in association with UMS have rarely been reported. In 2009, Linden et al. (30) reported a case of a female child with UMS whose mother, grandmother, and great-grandmother had hyperthyroidism; however, the child's thyroid function test demonstrated no marked variations. In 2008, Galazzi et al. (25) reported a case of a male with UMS, who had hyperthyroidism and also displayed *TSHR* mutations. In the present case, even though the child had hyperthyroidism; because he displayed increased thyroid autoimmune antibodies, it can be considered as immune thyroiditis and not a result of *TBX3* mutation.

In conclusion, the patient presented various symptoms of UMS; some were relatively mild and can be attributed to the type of mutation in *TBX3*. The TBX3 gene consists of seven exons, wherein exons 1, 2, and 3, and a part of exon 4 encode the conserved T-box domain. Located at the amino-terminus, the T-box mediates binding of the transcription factor to the DNA. Missense mutations within or upstream of the T-box domain are responsible for the loss of TBX3 transcriptional activity and are often associated with the most severe phenotypes (13). The patient's gene variant was not in this domain but was located in exon 6. Exon 6 encodes the activation domain, and exon 7 encodes the R1 domain. Studies have shown that the activation domain can activate transcription and that this activation function is regulated by R1 (33). The activation and R1 domains of *TBX3* in the child are affected by the deletion mutation, resulting in the appearance of UMS symptoms. However, because the mutation did not involve the T-box domain, his symptoms were relatively mild.

This study reports the genotype and phenotype of a child with UMS and analyzes the relationship between the phenotype and genotype based on the literature. Owing to the clinical heterogeneity of UMS, it is easily missed and misdiagnosed in clinical practice. This study provides insight for clinicians in diagnosing the disease. Even if the symptoms are mild, once they are consistent with some phenotypes of UMS, genetic tests may be considered to take in order to make the diagnosis clear. To our knowledge, the described mutation in *TBX3* has been reported for the first time, which expands the mutation spectrum of the gene.

## Data Availability

The original contributions presented in the study are included in the article/Supplementary Material, further inquiries can be directed to the corresponding author/s.
